# Alterations in metabolic pathways: a bridge between aging and weaker innate immune response

**DOI:** 10.3389/fragi.2024.1358330

**Published:** 2024-03-05

**Authors:** Zahra Saleh, Sara Mirzazadeh, Fatemeh Mirzaei, Kamran Heidarnejad, Seppo Meri, Kurosh Kalantar

**Affiliations:** ^1^ Department of Immunology, School of Medicine, Shiraz University of Medical Sciences, Shiraz, Iran; ^2^ Department of Bacteriology and Immunology and the Translational Immunology Research Program (TRIMM), The University of Helsinki and HUSLAB, Helsinki University Hospital, Helsinki, Finland; ^3^ Autoimmune Diseases Research Center, Shiraz University of Medical Sciences, Shiraz, Iran

**Keywords:** aging, innate immunity, metabolic pathways, immunometabolism, age-related diseases

## Abstract

Aging is a time-dependent progressive physiological process, which results in impaired immune system function. Age-related changes in immune function increase the susceptibility to many diseases such as infections, autoimmune diseases, and cancer. Different metabolic pathways including glycolysis, tricarboxylic acid cycle, amino acid metabolism, pentose phosphate pathway, fatty acid oxidation and fatty acid synthesis regulate the development, differentiation, and response of adaptive and innate immune cells. During aging all these pathways change in the immune cells. In addition to the changes in metabolic pathways, the function and structure of mitochondria also have changed in the immune cells. Thereby, we will review changes in the metabolism of different innate immune cells during the aging process.

## 1 Introduction

Aging is a complex process in which time-dependent progressive physiological changes result in impaired biological functions and decreased quality of life (López-Otín et al., 2013). The immune system is one of the major biological systems affected by aging. Age-related changes in the structure and function of the immune system components lead to an increase in the susceptibility of older people to infections, autoimmune diseases, and cancer, a decrease in response to vaccines, and low-grade chronic inflammation (inflammaging) in blood and tissues (Sadighi Akha, 2018). Inflammaging can be triggered by cellular senescence, accumulation of damaged self-debris, impaired autophagy, mitochondrial dysfunction, decline of protein hemostasis (proteostasis), and microbiota dysbiosis (Franceschi and Campisi, 2014; Sanada et al., 2018; Teissier et al., 2022). These events elicit constitutive production of pro-inflammatory cytokines like interleukin-6 (IL-6) and tumor necrosis factor alpha (TNF-α) (Bruunsgaard, 2006; Maggio et al., 2006). The continuous presence of age-related inflammatory responses may cause metabolic dysregulation; in return, the dysregulation can exacerbate inflammaging (Kim et al., 2020). Consequently, inflammaging is a notable risk factor for age-related diseases and death (Franceschi et al., 2000).

Immune cells encounter different metabolic demands based on their state (resting or activated) and tissue environment (different oxygen levels and nutrient accessibility) (O'Neill et al., 2016; Loftus and Finlay, 2016). Overall, six main metabolic pathways regulate the metabolism of immune cells: glycolysis, tricarboxylic acid (TCA) cycle, amino acid metabolism, pentose phosphate pathway, fatty acid oxidation (FAO), and fatty acid synthesis (O'Neill et al., 2016). Aging could affect each of these metabolic pathways in adaptive and innate immune cells. This could lead to changes in immune cell functions and alterations in the microenvironment (Sadighi Akha, 2018; Martin et al., 2021). Some of these will be mentioned below.

Based on different studies, T cells go through metabolic and epigenetic changes during aging. These include decreased glycolysis, mitochondrial biogenesis and one-carbon metabolism, while increasing reactive oxygen species (ROS) (Ron-Harel et al., 2018; Quinn et al., 2019; Quinn et al., 2020; Nian et al., 2021; Møller et al., 2022). Also, studies have revealed that B cells during aging upregulate Glut1 expression and increase glucose uptake, oxidative phosphorylation (OXPHOS), anaerobic glycolysis and FAO (Kurupati et al., 2019; Frasca et al., 2021; Frasca et al., 2022).

**FIGURE 1:**
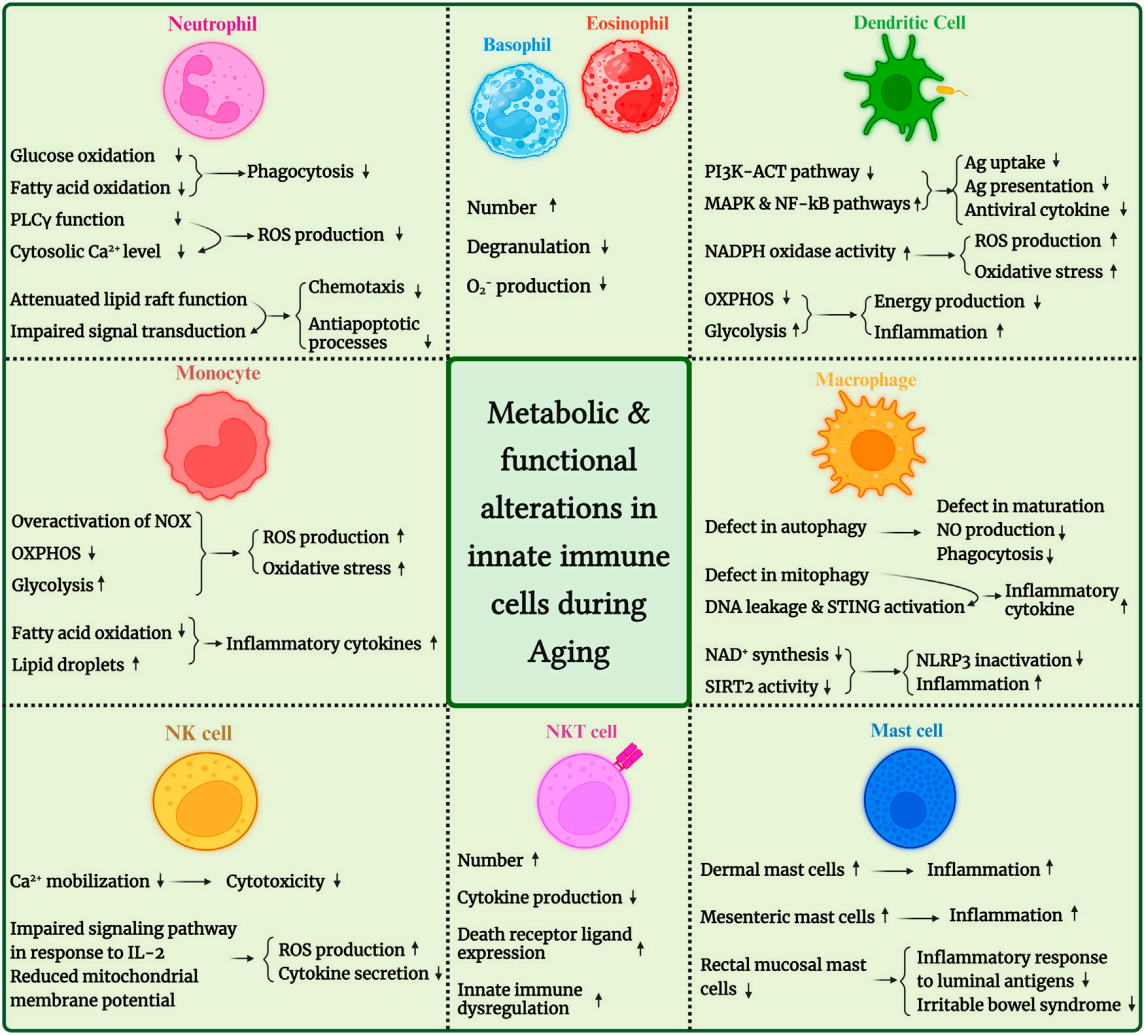
An overview of metabolic and functional alterations in innate immune cells during aging. PDK4, pyruvate dehydrogenase kinase 4; SIRT, sirtuin; ROS, reactive oxygen species; NLRP3: NLR family pyrin domain containing 3; STING, stimulator of interferon genes; IP3, inositol triphosphate.

Moreover, age-related changes in innate immune cells’ metabolic fitness have been evaluated in various studies. So, this mini-review will sum up the observations and reports about age-related changes in innate immune cell metabolism. We have illustrated these changes in Figure 1 for better understanding. We also provided an overview of age-associated changes in human and mouse innate immune cells in Table 1.

**TABLE 1:** An overview of age-associated changes in human and mouse innate immune cells.

Cell type	Age related changes in metabolism	Cellular dysfunction resulted from metabolic alteration	Organism	References
Neutrophil	Decreased glucose oxidation	Decreased phagocytosis	H & M	Richer et al. (2018), Schaffer and Kim (2018), Tappia et al. (2018), Bae et al. (2022), Miyazaki et al. (2022)
	Decreased fatty acid oxidation			
	Decreased PLCγ function	Decreased ROS production	H	Lipschitz et al. (1988), Wenisch et al. (2000), Gomez et al. (2008), Immler et al. (2018)
	Decreased cytosolic calcium levels			
	Attenuated lipid raft function	Decreased chemotaxis	H & M	Fulop et al. (2004), Tortorella et al. (2006), Panda et al. (2009), Fulop et al. (2014), Zhang et al. (2022)
	Impaired signal transduction	Decreased antiapoptotic processes		
Eosinophil & Basophil	NR	Increased number	M	Busse et al. (2007), Van Beek et al. (2018)
		Decreased degranulation	H	Mathur et al. (2008)
		Decreased superoxide anions production		
Dendritic cell	Decreased PI3K- ACT signaling pathway	Decreased antigen uptake	H & M	Agrawal et al. (2007a), Agrawal et al. (2009), Maletto et al. (2010), Gardner et al. (2017)
	Increased MAPK and NF-kB pathways	Decreased antigen presentation		
		Decreased antiviral cytokine production		
	Increased activity of NADPH oxidase enzyme	Increased ROS production	H & M	Linton and Thoman (2014)
		Increased oxidative stress		
	Decreased oxidative phosphorylation	Decreased energy production	H & M	Agrawal et al. (2007b), Chougnet et al. (2015), Rahmatpanah et al. (2019)
	Increased glycolysis pathway	Increased inflammation		
Monocyte	Increased activation of NADPH oxidase enzyme	Increased ROS production	H	Pence and Yarbro (2018), Saare et al. (2020)
	Decreased oxidative phosphorylation	Increased oxidative stress		
	Increased glycolysis pathway			
	Decreased fatty acid oxidation	Increased inflammatory cytokines production	H & M	Stahl et al. (2018)
Macrophage	Defect in autophagy	Defect in maturation	H & M	Stranks et al. (2015), Khalil et al. (2016), Zhong et al. (2022)
		Decreased NO production		
		Decreased phagocytosis		
	Defect in mitophagy	Increased inflammatory cytokines production	H & M	Li et al. (2020)
	Decreased NAD + synthesis	Decreased NLRP3 inactivation	H	Gounder et al. (2018), Cameron et al. (2019)
	Decreased SIRT2 activity	Increased Inflammation		
NK cell	Decreased Ca2+ mobilization	Decreased cytotoxicity	H	Dustin and Long (2010), Judge et al. (2020)
	Impaired signaling pathway in response to IL-2	Increased ROS production	H & M	Bae et al. (2019), Menees et al. (2021)
		Decreased cytokine secretion		
NKT cell	NR	Increased number	H & M	Shaw et al. (2013)
		Decreased TH1 type cytokine production	H	Shaw et al. (2013)
		Increased death receptor ligand expression		
		Decreased innate immune dysregulation	M	Inui et al. (2002), Dahlin et al. (2022)
Mast cell	NR	Increased dermal mast cells & Increased inflammation	H	Chatterjee and Gashev (2012)
		Increased mesenteric mast cells & Increased inflammation	H	Dunlop et al. (2004)
		Decreased rectal mucosal mast cells & Reduced inflammatory response to luminal antigens	H	Barbé-Tuana et al. (2020)

H, Human; M, Mouse; NR, None Reported.

## 2 Metabolic alterations in innate immune cells

### 2.1 Neutrophil

Neutrophils are the most abundant leukocytes in the human peripheral blood. They are essential in the first line of defense against invading microorganisms (Curi et al., 2020). The first step for killing microorganisms by the neutrophil is the engulfment of foreign particles by phagocytosis. This process is disrupted in aging because of low levels of taurine. Taurine acts as an antioxidant and plays a vital role in neutrophil phagocytosis. In both human and mice taurine exerts an important impact on energy metabolism. Taurine deficiency impaired complex I activation and subsequent an elevation occurs in NADH/NAD + ratio. This Inhibit the function of dehydrogenase enzymes and reduce glucose oxidation during glycolysis. Lower levels of taurine also reduce PPARα transcription factor and as a result suppress fatty acid oxidation. With these metabolic alterations a reduction occurs in the level of adenosine triphosphate (ATP) in the neutrophils of aged people. As a result, there is an increased energy demand or decreased energy production in these cells. These changes show that how taurine deficiency and subsequent detrimental changes in metabolism attenuate neutrophil function (Richer et al., 2018; Schaffer and Kim, 2018; Tappia et al., 2018; Bae et al., 2022; Miyazaki et al., 2022).

Another problem reported on neutrophils of aged people is related to a dysregulation of intracellular calcium influx. Investigations have revealed a decrease in intracellular calcium influx. This phenomenon has been attributed to a reduced phospholipase C-γ (PLCγ) function and significantly reduced generation of diacylglycerol (DAG) and inositol triphosphate (IP3). Subsequently, less calcium will be released through the IP3 sensitive calcium channel in the endoplasmic reticulum. Lower cytosolic calcium concentrations in stimulated aged neutrophils lead to a reduced phagocytic capacity and diminished ROS production (Lipschitz et al., 1988; Wenisch et al., 2000; Gomez et al., 2008; Immler et al., 2018). Taken together, aging manipulates metabolic activity of neutrophils in a way that attenuates the effector function of these cells.

Neutrophils in both aged people and mouse also seem to have dysfunctional signal transduction. Lipid rafts are part of the phospholipid bilayer membrane and are rich in cholesterol and phospholipid. They are essential for the regulation of signal transmission from different receptors. Lipid raft function may be attenuated in human-aged neutrophils because the physicochemical properties of neutrophil membranes alter with age. It has been found that the fluidity of the neutrophil membrane increases as a consequence of changes in membrane composition. So, the cholesterol content of the neutrophil membrane increases, while membrane phospholipid levels do not change. Moreover, lipid raft distribution is disorganized with aging, which reduces lipid raft accumulation and disrupts downstream signaling events in aged neutrophils (Fulop et al., 2004; Zhang et al., 2022). Thus, signaling through receptors such as granulocyte macrophage-colony stimulating factor (GM-CSF) or formyl-methionyl-leucyl-phenylalanine (fMLP) receptor, activation of the Janus kinase/signal transducer and activator of transcription (JAK/STAT), extracellular signal-related kinases 1 and 2 (ERK1/2), PLCγ/protein kinase C (PKC) and phosphoinositide 3-kinase (PI3K)/protein kinase B (Akt) pathways will be reduced. In turn, this leads to reduced superoxide generation, chemotaxis, and antiapoptotic processes (Tortorella et al., 2006; Panda et al., 2009; Fulop et al., 2014).

In conclusion it seems that age related changes in metabolism correlate with impairment in neutrophil functions. So that neutrophils in elderly individuals and mouse have problems in signal transduction, phagocytosis, energy utilization and production.

### 2.2 Eosinophil and basophil

Eosinophils and basophils are among polymorphonuclear cells (PMN) that play crucial roles in combating against parasitic infection (Wechsler et al., 2021). Very low evidence is available regarding age associated changes in the function and number of eosinophil and basophil. Higher number of eosinophils have been detected in aged mice (Busse et al., 2007). Analyzing age-related changes in eosinophil function demonstrate a reduction in eosinophil degranulation and superoxide anions production in response to stimuli in the older asthmatic patients (Mathur et al., 2008). The influence of aging on basophil was analyzed in one study and higher number of basophils was detected in bone marrow and spleen of older mice (Van Beek et al., 2018).

### 2.3 Dendritic cell

Dendritic cells (DCs) are crucial for activating and instructing T cells and creating a bridge between innate and adaptive immunity (Hilligan and Ronchese, 2020). These cells are characterized by their efficient conversion of internalized antigens into peptide-major histocompatibility complexes (p-MHC) required to orchestrate T-cell responses. Age-related changes in DC function have been examined in mice model by Gang Li and his colleagues. They observed an impaired capacity of aged DCs in migration, antigen uptake and presentation, as well as reduced costimulatory molecule expression. They concluded that age-associated defects in DC functions contribute to the impaired adaptive immune response against microbial pathogens (Linton et al., 2005; Li et al., 2012). Furthermore, aging is associated with changes in several signaling pathways in mice and human. Phosphorylation of AKT decreased in aged DCs and subsequently reduced induction of the PI3K pathway. Reduced phosphorylation of AKT in aged DCs may cause overactivation of mitogen-activated protein kinase (MAPK) and nuclear factor-kB (NF-kB) pathways. Impaired induction of interferon regulatory factor 1 (IRF-1) and IRF-7 have also been detected in aged DCs. Defects in these pathways negatively influence DC capacity in T cell activation. Decreased antigen phagocytosis, reduced migration, and impaired production of anti-viral cytokines are among the defects affecting functions in aged DC (Agrawal et al., 2007a; Agrawal et al., 2009; Maletto et al., 2010; Gardner et al., 2017). Prakash et al. investigated the effect of age on DC function. Their results indicated that monocyte-derived DCs from the aged individuals are impaired in their capacity to produce interferon (IFN)-I and IFN-III in response to the influenza virus. They also observed age-associated epigenetic changes in the chromatin structure, which could be linked to reduced gene expression and subsequently reduced cytokine production (Prakash et al., 2013).

In addition to the mentioned problems regarding the DCs during aging, it is worth to mention that reduced gene expression in the mitochondrial electron transport chain also occurs. Aged DCs in both human and mice exhibited reduced mitochondrial membrane potential, energy production, and baseline OXPHOS (Agrawal et al., 2007b; Chougnet et al., 2015; Rahmatpanah et al., 2019). Nevertheless, the expression of glucose and amino acid transporters is upregulated in DCs from aged subjects, which is a way to compensate for reduced energy generation. Moreover, glucose and amino acids are primary activators of mammalian target of rapamycin complex 1 (mTORC1) signaling. mTORC1 regulates anabolic processes and promotes glycolysis and secretory phenotype (many cytokines, chemokines, and growth factors promoting inflammation and tumor growth) in aged DCs. So, mTOR inhibition has increased longevity in mice and human (Papadopoli et al., 2019; Rahmatpanah et al., 2019).

In inflammatory cells, ROS is derived from the electron transport chain and produced by the action of the nicotinamide adenine dinucleotide phosphate (NADPH) oxidase enzyme. In DCs from elderly individuals and old mouse, ROS production is increased, while the activity of ROS converting enzymes is decreased. These changes promote ROS accumulation in aged DCs and consequently oxidation of proteins, carbohydrates, lipids, and nucleic acids, a process known as oxidative stress. Oxidized proteins accumulate and disrupt the function of aged DCs (Linton and Thoman, 2014). It can be concluded that overactivation of MAPK and NF-kB pathways, increased activation of NADPH oxidase enzyme in electron transport chain and subsequent accumulation of ROS and oxidation of macromolecules, reduced oxidative phosphorylation and increased glycolysis pathway are among the age-related changes occur in DC metabolism in both human and mouse and are followed by DC dysfunction.

### 2.4 Monocyte

Monocytes are circulating blood cells that make up 10% of peripheral leukocytes in humans and 4% in mice. They perform various immune effector functions, such as recognizing pathogens through Toll-like receptors (TLRs) and other pattern recognition receptors (PRRs) and then secreting pro-inflammatory cytokines, presenting antigens, assisting in tissue remodeling and wound healing, and producing anti-inflammatory cytokines and lipid mediators to help reduce inflammation (Guilliams et al., 2018). Monocyte classification is based on CD14 and CD16 expression. The population of human primary monocytes may be divided into three subsets: classical (CD14^high^/CD16^-^), intermediate (CD14^high^/CD16^+^), and non-classical (CD14^low^/CD16^+^) (Ong et al., 2018).

Monocytes are among the phagocytic cells of the innate immune system, which face many changes in aging, such as increased production of cytokines and inflammatory mediators, decreased phagocytosis, changes in the population of subsets and metabolic changes (De Maeyer and Chambers, 2021).

During aging, lower mitochondrial respiratory capacity and higher levels of ROS have been detected in classical monocytes from elderly individuals. Damaged mitochondria or increased activity of NADPH oxidase could be the deriver of elevation in ROS level (Pence and Yarbro, 2018; Saare et al., 2020). On the other hand, in aged monocytes, levels of pyruvate dehydrogenase kinase (PDK4), which is an inhibitor of pyruvate dehydrogenase (PDH), are increased. As a consequence, this causes a shift in the metabolic pathway from OXPHOS to lactate production. In addition, glucose uptake is increased in aged monocytes, which indicates more efficient glycolysis in aged monocytes (Saare et al., 2020). In contrast, one study indicated no variations in glycolysis between young and aged monocytes in *ex vivo* (Pence and Yarbro, 2019). The monocyte isolation method, along with other possible causes of variance like subject number, population demographics, age ranges, *etc.*, may contribute to these disparate results.

In aged monocytes, levels of enzymes that convert phosphatidylcholine to arachidonic acid are significantly decreased in the aged monocytes. As a result, the production of anti-inflammatory mediators is downregulated (Saare et al., 2020). According to one study, increased quantities of lipid droplets (LDs) were detected in the monocytes of older mouse and people, and this increase was linked to decreased FAO. Also, the pro-inflammatory phenotype of monocytes in older people may be caused by downregulated peroxisome proliferator-activated receptor (PPAR)-alpha. This was positively connected with LD accumulation and rising TNF-α concentration (Wang et al., 2021). In conclusion, glycolysis increased along with elevated in glucose uptake in aged monocytes while OXPHOS decreased and ROS increased. Furthermore, deposition of lipid droplets could contribute to the production of inflammatory cytokines during aging in monocytes.

Altogether, aging is associated with detrimental changes in the metabolism of monocytes. Mitochondrial dysfunction, overproduction of ROS, reduced OXPHOS, increased glycolysis and decreased FAO are among these metabolic alterations that adversely affect monocyte function.

### 2.5 Macrophage

Macrophages are essential for innate immunity because they serve as sentinels to combat infections, hasten the healing of wounds, and control the emergence of a particular acquired immune response (De Maeyer and Chambers, 2021).

In aged macrophages, autophagy is defective in both mouse and human (Stahl et al., 2018). According to a study, this defect in autophagy is due to increased hypermethylation in the promoter of autophagy-related 5 (ATG5) and microtubule-associated proteins 1A/1B light chain 3 B (LC3B) genes which promote the autophagic process. As a consequence, that causes a decrease in their expression in aged macrophages compared to young macrophages (Khalil et al., 2016). Consequently, the dysfunction of autophagy causes a decrease in the maturation of macrophages, nitric oxide (NO) production, and phagocytic properties (Stranks et al., 2015). In addition to autophagy, mitophagy, a kind of macroautophagy that deletes old and damaged mitochondria, is impaired in aged macrophages from mouse and human. Defects in mitophagy result from defects in mitochondrial polyubiquitination by PTEN-induced kinase 1 (PINK1)/Parkin as well as defects in lysosome biogenesis and function. Due to the defect in mitochondrial mitophagy in aged macrophages, mitochondrial DNA leaks into the cytosol during sterile damage and activates the cyclic GMP‐AMP synthase (cGAS)/stimulator of interferon genes (STING) pathway, promoting inflammatory cytokine production (Zhong et al., 2022).

In the context of metabolism, Nicotinamide adenine dinucleotide (NAD^+^) is an electron acceptor involved in OXPHOS, glycolysis, and many metabolic pathways. It is produced through the *de novo*, salvage, and Preiss-Handler pathways (Li et al., 2020). NAD^+^ decreased during aging in macrophages. Blocking the *de novo* NAD^+^ synthesis pathway in macrophages induces inflammatory phenotype, impairs OXPHOS, and increases ROS, which indicates the critical role of the *de novo* NAD^+^ synthesis pathway in controlling the metabolism of macrophages. Aged macrophages express less quinoline phosphoribosyl transferase (QPRT), as a critical enzyme in the *de novo* pathway compared to young macrophages. Consequently, decrease the *de novo* NAD^+^ synthesis pathway. This phenomenon increased inflammatory phenotype of macrophages and disruption of mitochondrial processes (Minhas et al., 2019). Also, it has been shown that the inflammatory macrophage depends on the production of NAD^+^ from the salvage pathway. Stimulation of macrophage with lipopolysaccharide (LPS) increases the critical enzyme in the salvage pathway. Boosting the salvage pathway is necessary to prevent DNA damage caused by ROS and maintain the inflammatory phenotype (Cameron et al., 2019). Furthermore, a study infers that aging macrophages shift their NAD^+^ metabolism from the *de novo* synthesis pathway to the salvage synthesis pathway (Pence, 2021).

The activity of Sirtuins can be impacted by alteration in NAD^+^ metabolism since they are NAD^+^-dependent deacetylases that control critical metabolic pathways in cells. Sirtuin 2 (SIRT2) deacetylates NLR family pyrin domain containing 3 (NLRP3) and inactivates the NLRP3 inflammasome in macrophages. Moreover, induction of the overexpression of SIRT2 in aged macrophages causes a decrease in the production of interleukin-1β (IL-1β) and the activation of NLRP3. In other words, one of the causes of inflammation can be related to the decrease in SIRT2 activity (He et al., 2020). SIRT3 is also an NAD^+^-dependent deacetylase that affects the function of mitochondrial proteins and OXPHOS. SIRT3 deacetylates the complex I of the electron transport chain. In addition, decreasing *de novo* NAD^+^ synthesis reduces SIRT3 activity, leading to impaired OXPHOS (Minhas et al., 2019). In conclusion, the decline in NAD^+^'s *de novo* synthesis pathway and changes in NAD^+^-dependent deacetylases, such as SIRT2 and SIRT3, play critical roles in macrophage function and age-related inflammation.

Defect in autophagy, defect in mitophagy, defect in NAD^+^ synthesis and NAD^+^ dependent deacetylases, and defect in OXPHOS are among the disturbance occurred in the metabolism of macrophages in old mouse and elderly individuals. These deleterious changes directly correlate with macrophage dysfunction.

### 2.6 NK cell

Natural killer (NK) cells are the primary defense lymphocyte against viral infection and represent 10%–15% of peripheral blood lymphocytes, which are responsible for antimicrobial response, adoptive immunity induction, and clearance of senescent cells (Gounder et al., 2018; Solana et al., 2018). In older people, decreased activity of NK cells is associated with more tumor prevalence, lower vaccination efficacy, accumulation of senescent cells, and infection susceptibility, which shorten survival (Miranda et al., 2018).

Release of lytic granules into the immune synapse (IS) is one of the NK cell cytotoxic events, which requires several checkpoints to perform the alteration of phosphoinositide molecules by PLC, calcium mobilization, and polarization of secretory lysosomes to the IS. Calcium ions (Ca2^+^) release is also required for mitochondria movement to the IS and fusion of lysosomes to the NK cell membrane. After interaction of a receptor with the relevant ligand, the cytoplasmic tail gets phosphorylated, recruits the PI3K, and provides two signaling pathways (Brauning et al., 2022). The first pathway is MAPK and NFκB pathway, which are activated by PI3K and PLC, respectively. The second one is the transformation of phosphatidylinositol 4,5-bisphosphate (PIP2) to phosphatidylinositol (Franceschi and Campisi, 2014; Sanada et al., 2018; Teissier et al., 2022)-triphosphate (PIP3), which leads to the production of IP3, the second messenger (Dustin and Long, 2010). IP3 is essential in this process by stimulating Ca2^+^ release from the internal store. IP3 production is shown to be significantly reduced in older humans’ NK cells (Mariani et al., 1998). Also, F. Borrego et al. reported that the Ca2^+^ mobilization in NK cells of older is decreased (Borrego et al., 1999). Therefore, reduced IP3 production and impaired Ca2^+^ mobilization might contribute to lower NK cell cytotoxicity in older humans (Brauning et al., 2022).

Decreased NK cell cytotoxic functions in elderly people and aged mouse are related to lower secretion of IFN-γ, downregulation of perforin and granzyme, and impaired NK cell cytotoxicity (NKCC) (Judge et al., 2020). IL-2 is a potent provoker of NK cytokine production and NKCC. It was shown that IL-2 could increase the production of IFN-γ and TNF-α from NK cells of older donors, but on a lower scale compared to younger donors (Menees et al., 2021; Brauning et al., 2022). Therefore, the signaling pathway in response to IL-2 might be impaired in older ones. Also, it revealed that increased secretion of IFN-γ from NK cells in response to IL-2 is associated with an increase in mitochondrial mass and membrane potential in peroxisome proliferator-activated receptor-𝛾 coactivator 1- α (PGC-1α)-dependent manner (Miranda et al., 2016). PGC-1α is a member of PGC-1 family, which acts as a transcriptional coactivator. These molecules form a protein complex by engaging nuclear receptors and transcriptional factors to regulate gene expression, which modulates mitochondrial biogenesis and respiratory function (Miranda et al., 2016). *D. Miranda* et al. indicated that purified NK cells from young donors have the potential to increase the PGC-1α by three folds after IL-2 stimulation. At the same time, there is an altered IL-2 signaling in NK cells of older donors, which affects mitochondrial functions and leads to increased production of ROS (Miranda et al., 2018). ROS is a molecule derived from superoxide (O2^−^), and eight regions within mitochondria can produce O2^−^ (Sena and Chandel, 2012).

In summary, aging affects NK cell metabolism in different aspects, which can lead to decreased activity. NK cells of older people exhibited an altered response to IL-2 by increasing ROS production instead of increasing expression of PGC-1α. Another alteration of the NK cell in older people is impaired IP3 production and Ca2+ mobilization. These changes lead to lower NK cell cytotoxicity and cytokine production.

### 2.7 NKT cell

NKT cells as a distinct subset of immune cells are characterized based on the expression of T cell receptor and also some NK cell expressing markers. These cells activate upon recognition of CD1d-lipid complex presented by different immune cell types. NKT cells play important roles in defense against cancer and microbial infections through cytokine production, direct cytotoxicity and induction of apoptosis via death receptor-ligand interaction (Chen and Liao, 2007; Fujii et al., 2013; Bae et al., 2019). Aging is associated with increase in the absolute number and percentage of NKT cells both in the circulation and lymphoid organs. In elderly individuals, NKT cells produce lower levels of TH1 cytokines. and express higher level of death receptor ligand (Plackett et al., 2004; Shaw et al., 2013). Studies performed on mouse model indicate that aging potentiate inflammatory response of NKT cells and promote innate immune dysregulation (Inui et al., 2002; Kawabata et al., 2008).

### 2.8 Mast cell

Mast cells are central players of allergy reaction and a variety of disease. These cells originate from hematopoietic stem cells in bone marrow and terminally differentiate in peripheral tissues. Mast cells are wildly distributed in different tissues including mucosal and connective tissues (Dahlin et al., 2022; Wagner et al., 2023). Human dermal mast cells increase in quantity with age. Dermis inflammation induced by both external and internal factors such as changes in temperature and air humidity and also mast cell chemoattractant produced in inflammatory process are involved in elevated levels of mast cells in derm (Petrov et al., 2013). Aging-associated inflammatory environment is accompanied by an increase in the number of mast cells located in close proximity to human mesenteric lymphatic vessels (Chatterjee and Gashev, 2012). Aging is associated with a reduction in the numbers of rectal mucosal mast cells. This may influence the low-grade inflammatory response to luminal antigens and contribute to the reduction of irritable bowel syndrome observed in older individuals (Dunlop et al., 2004).

As we discussed in this article, aging is associated with detrimental changes in the metabolism of innate immune cells. Some of these changes are shared among a number of innate immune cells. Reduced function of PLCγ and subsequent lower cytosolic calcium concentrations in aged neutrophils and NK cells, decreased oxidative phosphorylation and increased glycolysis pathway in aged dendritic cells and monocytes can be mentioned as an example of these common metabolic changes. However, inconsistent changes occur in the production of ROS in different innate immune cells. So that aging is accompanied with decreased ROS production in aged neutrophils, eosinophils and basophils. While increased levels of ROS have been detected in aged DCs, monocytes and NK cells. Several factors are involved in these inconsistent changes in ROS production. Lower level of taurine and subsequent disturbed function of electron transport chain complex I as well as disorganized lipid raft distribution and consequent impaired signal transduction are among the causes of reduced ROS production in aged neutrophils. Increased activity of NADPH oxidase enzyme in electron transport chain promotes more ROS production in aged DCs and monocytes. Furthermore, defect in IL2 signaling pathway is associated with reduced mitochondrial membrane potential and more ROS production in aged NK cells.

As a whole, common or inconsistent changes in the metabolism of innate immune cells contribute to multiple functional impairments. Defect in cytotoxicity, phagocytosis, antigen presentation, cytokine production and chemotaxis are among these functional disorders.

## 3 Consequences of aging on predisposition to diseases

As described in this review, metabolic alterations are one of the many mechanisms that lead to the malfunction of innate immune cells and the production of inflammatory mediators (inflammaging). Subsequently, inflammaging can contribute to the development of several age-related diseases, such as neurodegenerative diseases, rheumatoid arthritis, cancer, cardiovascular, and metabolic diseases (Barbé-Tuana et al., 2020).

Furthermore, mitochondria, as a vital organelle for metabolism, become defective during aging and contribute to diverse aging-related diseases. Impaired oxidative phosphorylation, mitophagy defect, mDNA release, accumulation of ROS, and oxidative stress increase the risk of heart failure, Alzheimer’s disease, osteoarthritis, osteoporosis, and aging-related macular degeneration (AMD) (Guo et al., 2022).

Also, during aging, the immune response to vaccines and pathogens declines due to the dysregulation of innate and adaptive immune systems. Several studies have declared the effect of aging on response to chronic viral infections and the effectiveness of vaccination. They observed the increased severity of viral and bacterial infections and impaired responses to vaccinations (Hainz et al., 2005; Tohme et al., 2011; Goldstein, 2012; Yang et al., 2016). Innate immune cells, especially DCs, are critical to triggering effective immune responses to vaccines. As mentioned in the DC section, the impaired capacity of aged DCs in migration, antigen uptake, and presentation contributes to the impaired adaptive immune response. Indeed, defects in several signaling pathways, like TLRs, negatively influence DC capacity in the expression of costimulatory molecules, cytokine production, and T cell activation. Altogether, aging-related dysfunction of innate immunity accounts for a decrease in elderly persons’ immune response to vaccines and pathogens (Panda et al., 2010; Chen et al., 2022).

## 4 Conclusion

Aging, as a physiologic progressive process, gives rise to different intrinsic and functional changes in immune cells; in return, these changes, over time, accelerate the aging process. Metabolic pathways possess a pivotal role in the survival and functions of immune cells by regulating energy demands and production. During aging, innate immune cells, as a first-line immunity and activator of adaptive immunity, face various metabolic alterations such as increasing glycolysis, decreasing FAO, and OXPHOS. These metabolic alterations lead to impaired phagocytosis, antiviral cytokine production, antigen presentation, and cytotoxicity of innate immune cells, besides increased production of inflammatory cytokines. Consequently, the immune cells cannot cope with infections and malignant cells, which make aged individuals susceptible to infectious diseases, cancer, and inflammatory diseases. Therefore, targeting these pathways in therapeutic strategies might be an approach to slow down the aging process.
